# Reliability and validation of an attitude scale regarding responsible conduct in research

**DOI:** 10.1371/journal.pone.0265392

**Published:** 2022-03-16

**Authors:** Samar Abd ElHafeez, Mohamed Salem, Henry J. Silverman

**Affiliations:** 1 High Institute of Public Health, Alexandria University, Alexandria, Egypt; 2 American University in Cairo, New Cairo, Egypt; 3 University of Maryland School of Medicine, Baltimore, Maryland, United States of America; Tabriz University of Medical Sciences, ISLAMIC REPUBLIC OF IRAN

## Abstract

**Background:**

Several studies reveal a problematic prevalence of research misbehaviors. There are several potential causes of research misconduct but ensuring that scientists hold attitudes that reflect norms of acceptable behaviors is fundamental.

**Aim:**

Our aim was to evaluate the psychometric properties (factor structure and reliability) of an “attitude” scale that we adopted from a questionnaire we previously used to investigate the prevalence of research misbehaviors in the Middle East.

**Methods:**

We used data from participants (n = 254) who were involved in our prior questionnaire study to determine the validity of an attitude scale that we adapted from this previous study. We performed exploratory factor analysis (EFA) to determine the factor structure of the attitude scale followed by measures of convergent and concurrent validity. We assessed reliability by computing the Cronbach’s alphas of each construct of the attitude scale.

**Results:**

EFA indicated that the attitude scale consists of two factors (constructs). Convergent validity was demonstrated by significant correlations of item-item and item-total. Correlation analysis revealed that the attitude constructs were significantly correlated with the Research Misbehavior Severity Score, thereby demonstrating concurrent validity. Cronbach’s alphas were greater than 0.75 for both constructs.

**Conclusion:**

We demonstrated a valid and reliable 20-item attitude scale with two factors related to “acceptability of practices in responsible conduct in research” and “general attitudes regarding scientific misconduct”. The use of a validated attitude scale can help assess the effectiveness of educational programs that focus on participants acquiring attitudes that are instrumental in responsible conduct in research.

## Introduction

Studies have documented the prevalence of research misconduct in Western [1–4] and in non-Western settings [5–7]. Regarding misconduct from the West, Martinson and colleagues surveyed US investigators’ self-report of their misbehaviors and demonstrated that falsification and plagiarism were 0.3% and 1.4%, respectively. The frequencies for other misbehaviors were above 5%; for example, "inappropriately assigning authorship credit" was 10.0%, and “dropping observations or data points from analyses based on a gut feeling” was 15.3% [2].

Studies from non-Western countries have shown a higher prevalence of research misbehaviors. Okonta and Rossouw revealed that 68.9% of Nigerian investigators admitted to having committed at least one of eight listed of types of scientific misconduct [6]. Felaefel and colleagues surveyed academics from several countries in the Middle East and showed that 59.4% of respondents self-reported committing at least one misbehavior [7]. These studies demonstrating extensive research misconduct serve to raise doubts regarding investigators’ integrity, which can erode society’s trust in science [8,9].

A variety of reasons can explain scientific misbehaviors. These include inadequate training, commercial and academic conflicts of interest, institutional failures of oversight [10], negative personality traits [11], failure of the organizational research climate to foster research integrity [12,13], and career and funding pressures [1,14].

Responsible conduct in research (RCR) may also be dependent on acquiring attitudes that reflect accepted norms regarding RCR [15–17]. Indeed, attitudes serve as a precondition "for someone to consider applying their learned knowledge or skills" [18].

Presently, only a few validated instruments that assess attitudes exist regarding RCR. Mavrinac and colleagues validated a questionnaire that included attitudes toward plagiarism, which represents only one construct of responsible misconduct [19]. The Scientific Misconduct Questionnaire—Revised (SMQ-R) represents a validated instrument, but it is narrow in scope as it measures clinical trial coordinators’ experiences with research misconduct [20]. Recently, Holm and Hofmann demonstrated the factor structure and reliability of the 2005 version of Kalichman’s “Survey 2: research misconduct” questionnaire [21]. From data obtained from three surveys among biomedical doctoral students in Scandinavia (2010–2015) these authors found that the 13-item scale to be reliable and factor analysis indicated that the overall scale can be divided into four subscales representing the following constructs: (a) general attitude to misconduct, (b) attitude to personal misconduct, (c) attitude to whistleblowing and (d) attitude to blameworthiness/punishment. In a follow-up study, these investigators used this questionnaire with postdoctoral students from Norway and showed that attitude scores reflective of acceptable norms of responsible research conduct were negatively correlated with research misconduct scores [22]. Further development of instruments that measures attitudes regarding responsible conduct in research are warranted. Our aim was to evaluate the psychometric properties of an attitude scale that we adapted from a questionnaire we had used in a previous study that investigated the prevalence of research misbehaviors of academics in the Middle East [7].

## Methods

### Development of the item pool of the “attitude” scale

From our previous study (7), we developed the item pool of the attitude scale section of the questionnaire from a review of the existing literature and previous questionnaires (deductive approach). These published resources provided an initial framework for the item pool that was expanded after discussions among the research team members. We next assessed content validity (CV) with an expert panel of five investigators with knowledge and expertise on RCR. We asked the experts to individually review and rate the items’ relevancy on a 4-point Likert scale (e.g., not relevant, somewhat relevant, quite relevant, very relevant). We deleted Items if two or more experts assessed items as being "not relevant”.

We conceptually hypothesized that 21 attitude questions from our previous questionnaire consisted of two constructs or factors. One construct represented “attitudes toward the acceptability of RCR practices”, which included 16 items divided in the following sub-constructs: a) circumventing research ethics regulations (3 items); b) data fabrication and falsification (4 items); c) plagiarism (3 items); d) authorship (3 items); and e) conflict of interest (3 items). The other postulated construct represented “general attitudes scientific toward misconduct” and consisted of five items. Table 1 shows the description of the item pool of each of these attitude constructs.

**Table 1:** Items in the attitude scales.

**Attitude Scale #1: Attitudes toward the acceptability of RCR practices**	
**Compliance with research ethics regulations**	
RE_1	Conducting research involving human subjects without prior approval from an Institutional Review Board or Ethics Committee
RE_2	Use of confidential information about research subjects without their authorization
RE_3	Not obtaining proper informed consent from participants
**Data Fabrication and Falsification**	
DFF_1	Making up research data
DFF_2	Changing research data without mentioning it
DFF_3	Dropping “outliers” without mentioning it
DFF_4	Selecting only those data that support your hypothesis
**Plagiarism**	
Plag_1	Publishing results that belong to someone else
Plag_2	Using someone else’s words or ideas without giving proper credit
Plag_3	Submitting a manuscript to a journal that you already published in another journal
**Authorship misconduct**	
Authorship_1	Giving authorship credit to someone who has not contributed substantively to a manuscript
Authorship_2	Denying authorship credit to someone who has contributed substantively to a manuscript
Authorship_3	Allowing your name to be put on papers to which you have made no reasonable contribution
**Conflict of interest**.	
COI_1	Awareness of a conflict of interest (e.g., you have a financial interest with a drug company, and you are conducting a study for them) and did not disclose it to either the ethics committee or a journal
COI_2	Compromising the rigor of a study’s design or methodology in response to pressure from a commercial or not for profit funding source
COI_3	Inappropriately altering or suppressing research results in response to pressure from a commercial or not for profit funding source
**Attitude Scale #2: General attitudes toward scientific misconduct**	
SM_1	I’m concerned about the amount of misconduct that occurs
SM_2	The responsibility for misconduct lies with the principal investigator only
SM_3	Investigators should report instances of research misconduct
SM_4	Investigators should declare conflicts of interest to the appropriate officials
SM_5	I should monitor my trainees’ work to ensure that they are developing into responsible researchers

### Data set for testing the psychometric properties

To test the validity and reliability of the “attitude” constructs, we used the data set from our previous study that was conducted between February 2015 to September 2015. We had distributed the questionnaire to a convenient sample of academics by a) sending a web link on SurveyMonkey® via a recruitment email, and b) distributing by "hand" to investigators at Cairo University. All questionnaires were returned anonymously. The language of the survey was in English.

We recruited participants from several universities in the Middle East located in Egypt, Lebanon, and Bahrain. Our target population included: 1) academic faculty; 2) individuals with master’s and PhD degrees and postdoctoral students; and 3) senior undergraduate students and individuals working in research positions (e.g., research assistants and technicians).

The questionnaire consisted of the following sections: 1) demographic data, including place of graduate school attended, previous research ethic training, and previous experience in conducting research; 2) respondents self-report of the frequency of their research misconduct ("Never," "Once or twice," or "Three or more"); 3) “attitudes of the acceptability of RCR practices”; and 4) “general attitudes toward scientific misconduct.”

Responses regarding the “acceptability of RCR practices” were measured with a five-point Likert scale ranging from "very acceptable" to "definitely unacceptable." Values of "1" to 5" were assigned to "very acceptable" to "definitely unacceptable." For each of the 16 items, the total scores were calculated by simple addition and ranged between 16–80.

Responses regarding the “general attitudes toward scientific misconduct” were measured with a five-point Likert scale ranging from "strongly agree" to "strongly disagree." Values of “1" to "5" were assigned to "strongly agree" to "strongly disagree". We reversed scored several questions that were worded opposite to the other questions. For each of the 5 items, a total score was calculated by simple addition and ranged between 5–25. We also calculated a “total attitude” score by simple addition of the scores of “attitudes toward the acceptability of RCR practices” and ‘‘general attitudes toward scientific misconduct”. Higher numbers for attitude scores are representative of accepted norms toward responsible conduct in research.

Regarding prevalence of misconduct, participants were asked to self-report how often they committed each type of misconduct by choosing either “never”, “once or twice” or “three or more times”. Our data showed that the latter response category exhibited small cell frequencies in the range of 3–5% of the total responses. To ensure meaningful categories with sufficient data for analysis, we transformed the respondents’ self-report of 16 different research misconducts into dichotomous responses: "never" and "one or more times” [23]. The specific misbehaviors are listed in Table 2 of our original publication [11]. We calculated a “Research Misconduct Severity Scale” (RMSS) similar to the method used by previous investigators [11,15]. To construct the RMSS, each misconduct item was assigned a value of “0” if respondents did not self-report the misconduct and a value of “1” if they self-reported the misconduct at least once in the last three years. To compute the RMSS, items related to fabrication and falsification and plagiarism were each given a weight value of 3 (7 items), items related to “circumventing research ethics regulations” and “conflict of interest" were each given a weight of 2 (6 items); and items regarding “authorship" were given a weight of 1 (3 items) [11]. The total RMSS score (16 items) ranged between 0–36 points. Higher numbers represent greater severity of research misconduct.

**Table 2:** Baseline characteristics of the study population N = 254.

	Number	Percentage
**Gender** *		
Male	96	37.9
Female	157	62.1
**Nationality**		
Egyptian	183	72.1
Lebanese	29	11.4
Bahraini/Others	42	16.5
**Research position**		
Academic Faculty	121	47.6
Master, PhD, postdoctoral	133	52.4
**Highest Degree Earned**		
Not graduated	15	5.9
BA/BSc	49	19.3
MSc/MPH/other degree	76	29.9
MD/PhD	114	44.9
**Prior training on research ethics**		
No	108	42.5
Yes	146	57.5
**Previous experience in conducting research**		
**Yes**	209	82.3
**No**	45	17.7
**Place of Graduate School Attended**		
Not yet graduated	15	5.9
North America (Canada/USA)	19	7.5
European Union/UK	27	10.6
Middle East/North Africa	190	74.8
Others (Russia, Japan, and Sub-Saharan Africa)	3	1.2

*One participant failed to report gender.

### Psychometric evaluation of the attitude scale

We assessed the psychometric properties of our “attitude” scale by investigating its construct validity and its reliability.

#### Construct validity

Construct validity represents the extent to which an instrument assesses a construct of concern. Construct validity can be demonstrated by evidence of content validity, face validity, structural or factorial validity as well as divergent, convergent and concurrent related validities. If these measures of construct validity are deficient, it will be difficult to interpret results from the questionnaire and inferences cannot be made regarding predictors of a behavior domain.

#### Factorial validity

Exploratory Factor Analysis (EFA) identifies the structure/dimensionality of observed data to reveal the underlying constructs that give rise to observed phenomena. To determine the factor structure of the attitude scale, an EFA was used to identify the underlying factors/constructs of our set of 21 attitude items. A “factor” represents a collection of the items that have similar patterns of responses to create a construct. The resulting factor structure would help confirm our *a priori* assumptions about the relationships among the items in each of our hypothesized constructs. EFA evaluates construct validity via two functions: it identifies the factor structure and the number of factors or constructs that underlie a set of variables, (i.e., the questionnaire items) and determines as to whether the factors are uncorrelated with each other [24].

Before doing the EFA, we assessed factorability with both the Kaiser-Meyer-Olkin Index (KMO) test and Bartlett’s test of sphericity. Kaiser-Meyer-Olkin (KMO) is a measure of sampling adequacy. The KMO statistics range from 0 to 1 with values closer to 1 denoting greater adequacy of the factor analysis (KMO ≥ 0.6 low adequacy, KMO ≥ 0.7 medium adequacy, KMO ≥ 0.8 high adequacy, KMO ≥ 0.9 very high adequacy). Bartlett’s test of sphericity determines whether the variables are correlated in an identity matrix; a significant p-value associated with this test (e.g., < 0.05), indicates that factorial analysis can be used [25]. To perform the EFA, we used the Principal Line axis factoring with Promax oblique rotation, which leads to the calculation of the factor loadings for each question item [26].

We then determined the number of factors to retain (i.e., to determine how many factors account for most of the variance of the original observed variables) based on three procedures: the Eigenvalue (>1) criteria; parallel analysis [27]; and a scree plot.

An Eigenvalue measures the amount of variation in the total sample accounted for by each factor and is determined by the sum of the squared factor loadings for that factor divided by the number of variables. Factors with Eigenvalue >1 are considered significant. In a scree plot, the Eigenvalues are plotted against the factors and the number of factors to retain is determined by the data point above the point of inflexion in the scree plot [28].

The identification of a group of questionnaire items that belongs to a “factor” is achieved through a process of “factor loading”, which shows the degree to which a question item loads or correlates with the factor [29]. There are rules to determine whether an item “loads” in a meaningful way on a factor [24]. The process of exploratory factor analysis results in the smallest and most compatible number of underlying factors from a larger set of initial variables on a questionnaire.

Question items with high factor loadings (a cut-off value of 0.40) are associated with the distinct factor [30]. Items with factor loadings below 0.40 are considered inadequate as they contribute <10% variation of the latent construct measured. Hence, it is often recommended to retain items that have factor loadings of 0.40 and above. Items should also not cross-load on more than one single factor. To summarize, Items that cross-load or that appear not to load uniquely on an individual factor are deleted, which reduces the number of questionnaire items for that construct.

#### Divergent validity

We next calculated the correlation between each of the factors (inter-factor correlation matrix) to determine divergent validity Correlation coefficients between any two factors that demonstrates statistically significant differences and is less than 0.70 confirms that each factor represent a distinct entity from the other factors [31]. This procedure confirms divergent validity. Essentially, measures of constructs that theoretically *should* be related to each other are determined to be related to each other *and* measures of constructs that theoretically should not be related to each other are determined not related to each other (that is, one should be able to discriminate between dissimilar constructs.

#### Convergent validity

We assessed convergent validity by determining the **inter-item** and **item-to-total correlations**, which are used to examine existence of relationships between individual items in a construct.

Inter-item correlation examines the extent to which items on a scale are assessing the same content. Items with very low item-to-total correlations provides evidence that the item is not measuring the same construct measured by the other items in the factor and may be deleted [24,32]. item-to-total correlation examines the extent to which items in a factor are correlated with the total score that is calculated from all items in the factor.

Demonstration of convergent validity provides further evidence of construct validity.

#### Concurrent validity

We also assessed concurrent validity as an indicator of construct validity. Concurrent validity represents the extent to which one measurement is backed up by a related measurement obtained at about the same point in time. We sought to demonstrate concurrent validity by calculating the correlation between each of the attitude scales (‘‘acceptability of RCR practices”, “general attitude toward research misconduct” and the combined attitude scale) with the RMSS score [33].

#### Reliability analysis

To assess reliability, we calculated Cronbach’s alphas for each construct of the attitude scale: "attitudes to acceptability of RCR practices” and “general attitudes regarding scientific misconduct”. As a rule of thumb, a Cronbach’s alpha of .70 to .80 is considered respectable for a scale for research use and an alpha more than .80 is considered very good [34].

#### Predictors of attitudes

We used multiple linear regression analysis to assess the predictive ability of the different independent criteria (demographics and data regarding previous ethics training and research experience) to discriminate between individuals regarding their attitudes toward research misconduct. We built three models to identify the predictors of the construct “attitudes of the acceptability of RCR practices”, ‘‘general attitude toward research misconduct” and the “combined attitude score”.

We performed all statistical analyses were done using SPSS version (21). All variables with p<0.05 are considered significant predictors.

#### Ethics

Ethics approval was obtained from the respective research ethics committees in Bahrain, Lebanon, and Egypt to perform the original survey study. We obtained ethics approval to perform secondary analysis of the original data set from the University of Maryland, USA (HP-00094812).

## Results

### Characteristics of the participants

We obtained completed surveys from 278 respondents of whom 212 were from universities in Egypt, 33 attended Royal College of Surgeons in Ireland in Bahrain, and 33 were from Ain Wazein Hospital in Lebanon.

For our analysis investigating the construct validity and reliability of our “attitude” scale, we used the data from the participants (n = 254) who completed the questionnaire beyond the “attitudes” questions. Ages ranged between 18 to 73 years and the mean age was 36 years, SD ± 12 years. Table 2 shows the baseline characteristics of our sample. More than 60% of participants were females (62.1%); the majority was of Egyptian nationality (72%). Almost one half (47.6%) represented academic faculty. One fourth (25.2%) had earned their Masters (MSc/MPH) while 44.9% had MD/PhD degree. There were 7.5% who attended faculties in North America, 10.6% in EU/UK, and 74.8% in the Middle East or North Africa. More than half (57.5%) of the respondents indicated they had received ethics training and 82.3%reported previous experience in research.

### Descriptive statistics of participants’ responses

Table 3 shows the results of the participants’ responses regarding the “acceptability of RCR practices” and the “general attitudes toward scientific misconduct” constructs. For the former construct, the percentages of ‘acceptability to the different items (very acceptable and acceptable) ranged from 4.3% for “publishing results that belong to someone else” item to 9.0% for ‘‘Selecting only those data that support your hypothesis” item. For the “general attitudes toward misconduct” construct; most of the study participants (85.6%) strongly agreed or agreed that” Investigators should report instances of research misconduct” and 35.8% strongly agreed or agreed that “the responsibility for misconduct lies with the principal investigator only”.

**Table 3:** Frequency responses to the two “attitude” scales among the study population.

	Attitudes toward acceptability of RCR practices				
RCR Practices	Very Acceptable N (%)	Acceptable N (%)	Neutral N (%)	Unacceptable N (%)	Very Unacceptable N (%)
RE_1: Conducting research involving human subjects without prior approval from an REC	15(5.9)	7(2.8)	31(12.2)	80 (31.5)	121(47.6)
RE_2: Use of confidential information about research subjects without their authorization	11(4.3)	7(2.8)	16(6.3)	73(28.7)	147(57.9)
RE_3: Not obtaining proper informed consent from participants	10(3.9)	6(2.4)	25(9.8)	85(33.5)	128(50.4)
DFF_1: Making up research data	9(3.5)	8(3.1)	8(3.1)	69(27.2)	160(63.0)
DFF_2: Changing research data without mentioning it	6(2.4)	7(2.8)	12(4.7)	81(31.9)	148(58.3)
DFF_3: Dropping “outliers” without mentioning it	8(3.1)	6(2.4)	28(11)	99(39.0)	113(44.5)
DFF_4: Selecting only those data that support your hypothesis	10(3.9)	13(5.1)	39(15.4)	78(30.7)	114(44.9)
Plag_1: Publishing results that belong to someone else	8(3.1)	3(1.2)	6(2.4)	63(24.8)	174(68.5)
Plag_2: Using someone else’s words or ideas without giving proper credit	7(2.8)	6(2.4)	4(1.6)	84(33.1)	153(60.2)
Plag_3: Submitting a manuscript to a journal that you already published in another journal	8(3.1)	6(2.4)	11(4.3)	85(33.5)	144(56.7)
Authorship_1: Giving authorship credit to someone who has not contributed substantively to a manuscript	9(3.5)	9(3.5)	30(11.8)	102(40.2)	104(40.9)
Authorship_2: Denying authorship credit to someone who has contributed substantively to a manuscript	10(3.9)	3(1.2)	3(1.2)	75(29.5)	163(64.2)
Authorship_3: Allowing your name to be put on papers to which you have made no reasonable contribution	6(2.4)	12(4.7)	20(7.9)	92(36.2)	124(48.8)
COI_1: Awareness of a conflict of interest (e.g., you have a financial interest with a drug company, and you are conducting a study for them) and did not disclose it to either the ethics committee or a journal	6(2.4)	6(2.4)	13(5.1)	94(37.0)	135(53.1)
COI_2: Compromising the rigor of a study’s design or methodology in response to pressure from a commercial or not for profit funding source	7(2.8)	4(1.6)	12(4.7)	96(37.8)	135(53.1)
COI_3: Inappropriately altering or suppressing research results in response to pressure from a commercial or not for profit funding source	6(2.4)	5(2)	11(4.3)	84(33.1)	148(58.3)

	**General attitudes toward scientific misconduct**				
**Attitudes**	Strongly agree	Agree	Neutral	Disagree	Strongly Disagree
SM_1: I’m concerned about the amount of misconduct that occurs	63(24.8)	120(47.2)	47(18.5)	15(5.9)	9(3.6)
SM_2: The responsibility for misconduct lies with the principal investigator only	24(9.4)	67(26.4)	24(9.4)	92(36.2)	47(18.5)
SM_3: Investigators should report instances of research misconduct	96(37.8)	120(47.8)	22(8.7)	4(1.6)	12(4.8)
SM_4: Investigators should declare conflicts of interest to the appropriate officials	95(37.4)	122(48.0)	25(9.8)	3(1.2)	9(3.6)
SM_5: I should monitor my trainees’ work to ensure that they are developing into responsible researchers”	81(31.9)	87(34.3)	66(26.0)	11(4.3)	9(3.6)

Table 4 shows the descriptive statistics of each “attitude” item of the questionnaire and the extent of acceptability (very acceptable and acceptable) and agreement (strongly agree and agree). For the attitudes regarding “acceptability of RCR practices”, the mean ranged from 4.07 to 4.49 and standard deviations were from 0.86 to 1.11. The means of the items of ‘‘general attitudes scientific misconduct” ranged from 1.78 to 3.24 and standard deviation were between 0.77 and 1.94.

**Table 4:** Descriptive statistics (mean ± SD) of the different items of the attitude questionnaire.

Variable	Mean ± SD
**Attitudes toward the acceptability of RCR practices**	
RE_1	4.12±1.11
RE_2	4.33±1.02
RE_3	4.24±0.99
DFF_1	4.43± 0.96
DFF_2	4.41±0.89
DFF_3	4.19±0.95
DFF_4	4.07 ± 1.08
Plag_1	4.54 ± 0.86
Plag_2	4.46 ±0.87
Plag_3	4.38 ± 0.92
Authorship_1	4.11± 0.99
Authorship_2	4.49±0.91
Authorship_3	4.24± 0.96
COI_1	4.36 ±0.87
COI_2	4.37±0.87
COI_3	4.43±0.86
**General attitudes toward scientific misconduct**	
SM_1	2.09±0.89
SM_2	3.24±1.28
SM_3	1.81±0.85
SM_4	1.78 ± 0.77
SM_5	3.06±1.94

For the “attitudes of the acceptability of RCR practices” construct, the percentages of (very unacceptable or unacceptable) ranged from 93.7% for ‘Denying authorship credit to someone who has contributed substantively to a manuscript” to 81.1% for “Giving authorship credit to someone who has not contributed substantively to a manuscript”.

For the “general attitudes toward scientific misconduct” construct; more than two-thirds of the study participants either (strongly agreed or agreed) that ‘‘I’m concerned about the amount of misconduct that occurs”, ‘‘Investigators should report instances of research misconduct”, and “Investigators should declare conflicts of interest to the appropriate officials”; almost two-thirds either (strongly agreed or agreed) that ‘‘I should monitor my trainees’ work to ensure that they are developing into responsible research”. Slightly more than one-third (35.8%) either, (strongly agreed of agreed) that the ‘‘The responsibility for misconduct lies with the principal investigator only”.

### Construct validity

#### Exploratory Factor Analysis (EFA)

We determined the factorability of the attitude scale. The Kaiser–Meyer–Olkin measure of sampling adequacy was 0.944, which is above the recommended value of 0.60, and the Bartlett’s test of sphericity was found to be highly significant (*p* < 0.001). The results indicate that the data is suitable for factor analysis.

**Fig 1:**
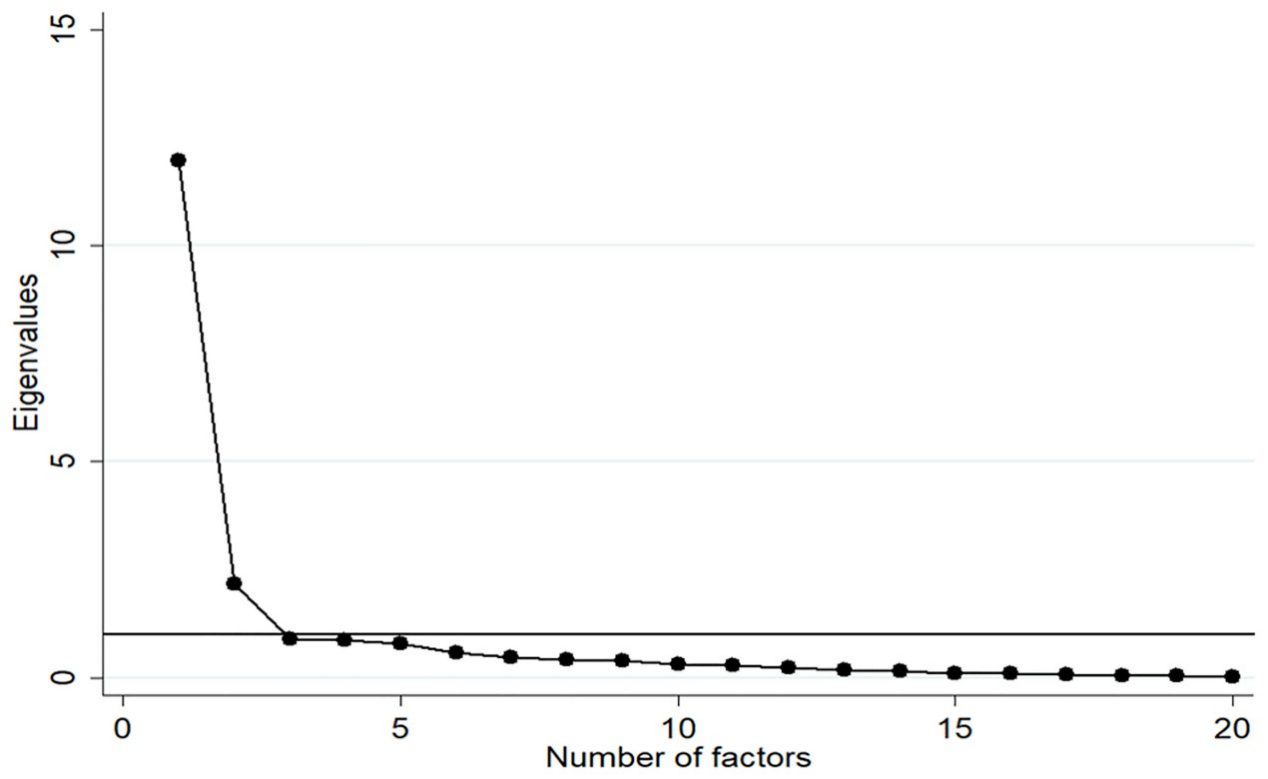
Scree plot for determining the numbers of factors extracted.

To decide how many of the factors to retain from the EFA, we identified that there were two factors with an Eigenvalue > 1. We confirmed this number of factors by parallel analysis and the scree plot which is shown in Fig 1. In the scree plot, the number of eigenvalues is on the y-axis and the number of factors on the x-axis. The “elbow” of the graph where the eigenvalues seem to level off Is indicated by a horizontal line parallel to x axis. The number of factors to the left of this point (or above the line) indicates that two factors should be retained. This analysis confirms that the two-factor solution was the best for the EFA analysis. The result also confirms our hypothesis regarding the number of constructs within the entire attitude scale. Subsequently, we performed the EFA with the two-factor model.

Using the Principal axis factoring with Promax oblique rotation, we calculated the factor loadings of the 21 items of the questionnaire. Table 5 shows the results of the EFA. We included items with loadings greater than or equal to 0.4 in the final EFA model. We deleted the item: “The responsibility for misconduct lies with the principal investigator only” as it loaded only with a value of 0.163. The final EFA included 20 items for the two factors. The two factors together explained 71.242% of the model cumulative variance.

**Table 5:** Factor loadings of the different items of the attitude scales: “Attitudes toward the acceptability of RCR practices” and “general attitude toward scientific misconduct”.

	Factor 1 (Attitudes toward the acceptability of RCR practices)	Factor 2 (General attitudes toward scientific misconduct)
	**Factor 1**	
RE_1	**.777**	-.035
RE_2	**.841**	-.079
RE_3	**.820**	-.039
DFF_1	**.878**	.021
DFF_2	**.908**	-.013
DFF_3	**.804**	-.015
DFF_4	**.790**	.026
Plag_1	**.917**	.045
Plag_2	**.918**	.037
Plag_3	**.860**	.071
Authorship_1	**.817**	.033
Authorship_2	**.909**	.013
Authorship_3	**.855**	-.003
COI_1	**.861**	-.041
COI_2	**.889**	-.010
COI_3	**.885**	.005
		**Factor 2**
SM_1	.031	**.410**
SM_3	.012	**.602**
SM_4	-.097	**.766**
SM_5	.075	**.709**
**% of variance**	61.005	10.236
**Cumulative %**	61.005	71.242

The inter-factor correlation between the two factors determined from the EFA was 0.263, which confirms divergent validity between the two factors.

#### Convergent validity

Table 1 (a) and 1(b) in S1 File show that the inter-item correlation of both constructs; “attitudes toward acceptability of RCR practices” and ‘‘general attitudes toward research misconduct’ were significant (p<0.001). These results demonstrate that the items in each factor are well related to each other and hence, are suitable to for measuring the same construct.

Table 2 in S2 File shows that the item-total correlations of “attitudes toward acceptability of RCR practices” and ‘‘general attitudes toward research misconduct” were significant (i.e., with each other); p<0.001. This signifies that every item of each factor is consistent (or correlates well) with the overall scale, which is additional evidence that all items in each factor represents a valid construct.

#### Concurrent validity

**Fig 2:**
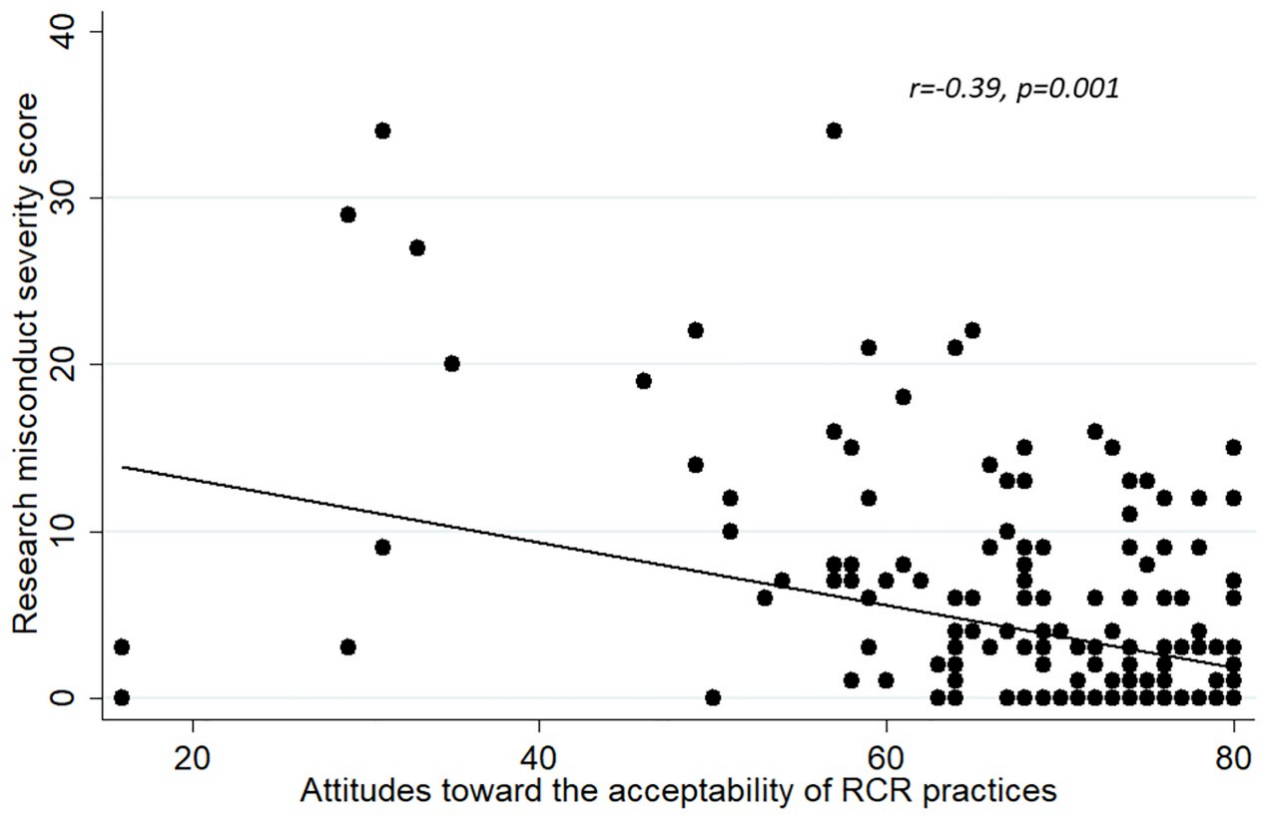
Correlation between the scores of the “attitudes toward the acceptability of RCR practices” and the Research Misconduct Severity Score.

**Fig 3:**
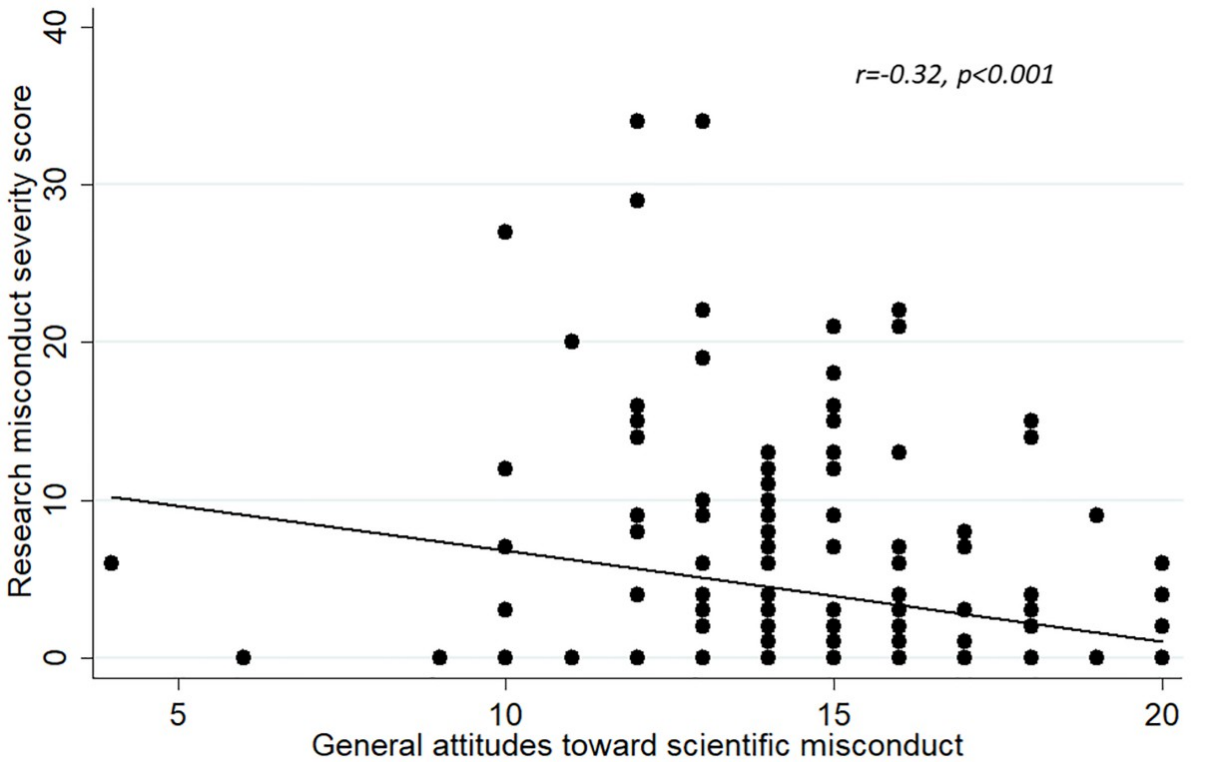
Correlation between the scores of the “general attitudes toward scientific misconduct” and the Research Misconduct Severity Score.

**Fig 4:**
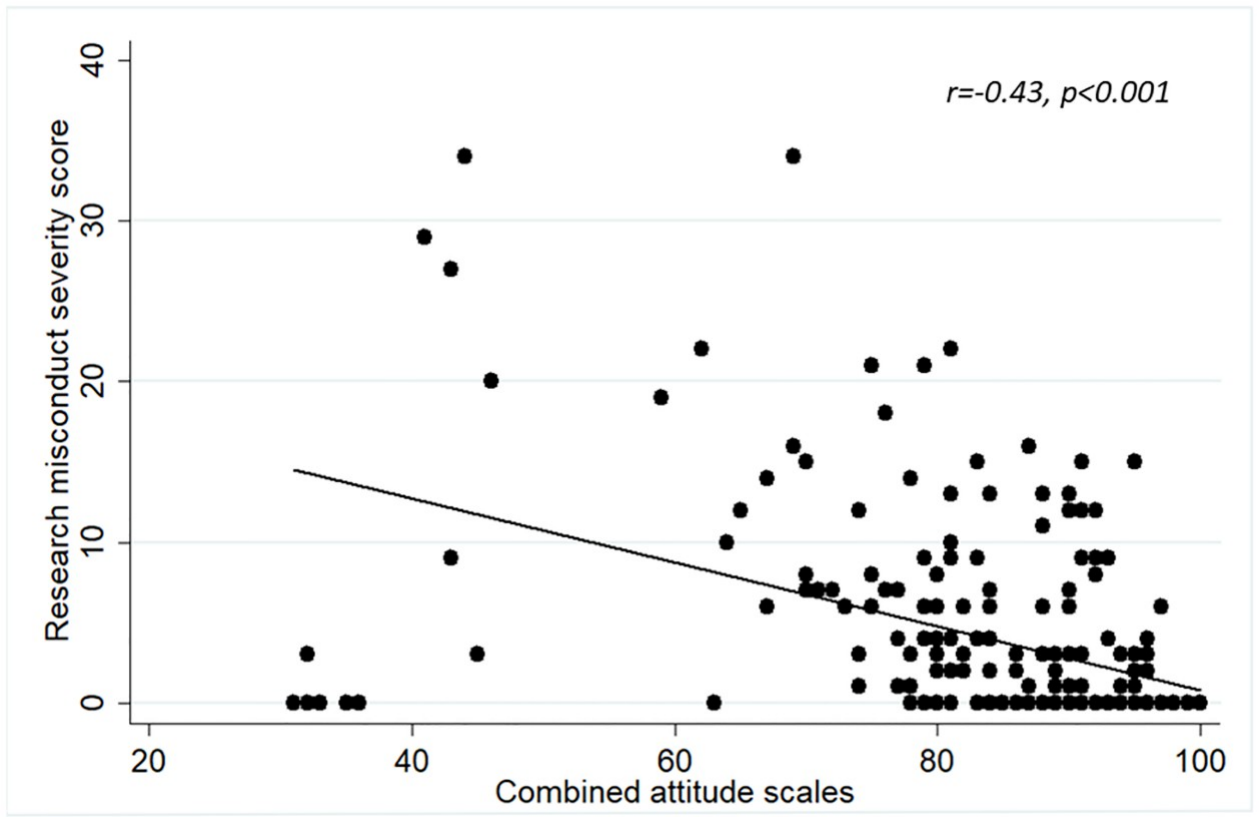
Correlation between the scores of the combined attitude scale and the Research Misconduct Severity Score.

Figs 2–4 show that the correlations between the individual total scores of each of the attitude constructs (individually and when combined) and the prevalence of the RMSS score. In each case, the “attitude” construct was significantly inversely correlated with the RMSS score. The more the respondents’ attitudes were according to acceptable norms of scientific conduct, the lower the RMSS score. As this result is expected, it further shows that the attitude scales represent valid instruments for measuring attitudes.

#### Predictors of attitudes

Table 6 shows the predictors of the three “attitude” scales. Participants who held a graduate degree, either BA/BSc or MSc/MPH/other degree, was significantly associated with higher scores for the “attitudes toward the acceptability of RCR practices”, ‘‘general attitudes toward research misconduct” and the combined attitude scale compared to those who had not graduated (p<0.05). Participants who held a MD/PhD degree was significantly associated with higher scores only for the ‘‘general attitudes toward research misconduct” scale compared to those who had not graduate (p<0.05).

**Table 6:** Predictors of the attitude scales (“attitudes toward the acceptability of RCR practices”, “general attitudes toward research misconduct”, and the “combined attitude scale”).

Variable	Attitude to the acceptability of RCR practices AOR* (95% CI), p-value	General attitude to research misconduct AOR* (95% CI), p-value	Combined attitude scale AOR* (95% CI), p-value
Age	0.07 (-0.12–0.27), p = 0.46	0.03 (-0.01–0.07), p = 0.09	0.09 (-0.11–0.30), p = 0.38
**Gender**			
Male	1	1	1
Female	0.97 (-2.57–3.98), p = 0.67	-0.14 (-0.80–0.53), p = 0.68	1.06 (-2.57–4.68), p = 0.57
**Nationality**			
Bahraini/Other	1	1	1
Egyptian	-1.16 (-6.04–3.72), p = 0.64	-0.68 (-1.63–0.28), p = 0.17	-1.87 (-7.07–3.34), p = 0.48
Lebanese	0.17 (-6.46–3.72), p = 0.64	-0.73 (-2.04–0.57), p = 0.27	0.25 (-6.86–7.37), p = 0.94
**Research position**			
Academic Faculty	1	1	1
Master, PhD, postdoctoral,	2.30 (-2.35–6.95), p = 0.33	-0.82 (-1.74–0.100), p = 0.08	1.51 (-3.51–6.53), p = 0.55
**Degree**			
Not yet graduated	1	1	1
BA/BSc	10.72 (3.15–18.28), p = 0.006	1.99 (0.52–3.47), p = 0.008	12.85 (4.81–20.90), p = 0.002
MSc/MPH/other degree	10.41 (2.48–18.34), p = 0.01	2.29 (0.75–3.83), p = 0.004	12.95 (4.56–21.34), p = 0.003
MD/PhD	6.00 (-2.89–14.90), p = 0.19	2.11(0.38–3.85), p = 0.02	8.62 (-0.84–18.08), p = 0.07
**Research ethics training**	3.42 (0.13–6.70), p = 0.04	0.63 (-0.02–1.28), p = 0.06	3.86 (0.33–7.38), p = 0.03
**Previous research experience**	1.78 (-3.20–6.76), p = 0.48	-0.69 (-1.66–0.29), p = 0.17	0.81(-4.50–6.12), p = 0.76

*AOR: Adjusted odds ratio.

Prior training in research ethics was significantly associated with higher scores on the “**Attitude to the acceptability of RCR practices” (p = 0.04) and to the** “combined attitude scale” (p = 0.03).

### Reliability analysis

The Cronbach’s alpha value was 0.975 for the 16 items in the “attitudes toward the acceptability of RCR practices” scale. The Cronbach’s alpha value was 0.754 for the four items in the “general attitudes toward research misconduct”. These values demonstrate a level of reliability that is respectable and acceptable, respectively.

## Discussion

We were able to determine the psychometric properties of an attitude scale that we adopted from a questionnaire we used in a previous study. Our factor analysis showed that the item pool of attitudes can be divided into two factors, each indicative of different constructs related to scientific research misconduct. One factor can serve as a valid and reliable measure of attitudes toward the acceptability of practices in responsible conduct in research while the other can serve as a valid and reliable measure of general attitudes toward research misconduct.

The significance of having a validated attitude instrument relies on the work of social scientists who demonstrated the significance of attitudes toward behavior when they theorized that individuals’ intentions to engage in certain behaviors is the best predictor of those behaviors. Ajzen’s and Fishbein’s theory posits that two components can predict intentions, which in turn predict behaviors [35]. One component is the person’s attitude toward the act in question. The other component measures the person’s perceptions of what other people expect him or her to do (subjective norms) and the motivation to comply with those expectations. Subjective norms represent the perceived social pressures to engage or not to engage in a behavior, thereby giving importance to ethical climate of the organization or region within which individuals are exposed. Hence, the overall theory of behaviors consists of three determinants: attitudes, subjective norms, and intentions. Gorsuch and Ortberg showed that the inclusion of a component of moral obligation added significantly to those of attitudes and subjective norms in predicting behaviors [36].

In our study, we demonstrated correlations between participants’ attitudes (both attitude constructs, individually and when combined) and their self-reported misconduct as measured by the RMMS. Based on the above-mentioned theory between attitudes and behaviors, this result is expected and provides further evidence of the construct validity of our attitude scales. To be sure, it is not possible from just the correlation analysis to determine the direction of any causal link between attitudes and behaviors, that is, attitudes might influence behavior or that behavior changes attitudes. Furthermore, even assuming from the correlation analysis that there is a direct influence of attitudes on misbehaviors, having “correct” attitudes while necessary, are insufficient predictors of behavior, as subjective norms are also important.

These results have implications for developing teaching strategies that aim to instill the appropriate attitudes as well as discussing the proper norms regarding responsible behaviors in research. Traditionally, RCR education have emphasized learning outcomes that mainly reflect Bloom’s cognitive and psychomotor domains [37]. Investigators have demonstrated statistically significant but modest outcomes in both domains [38,39]. Conversely, Bloom’s affective domain (representing characteristics such as “interests, attitudes, appreciations, values, and biases” [40] is more congruent to the behavioral model espoused by Ajzen and Fishbein [35] and Gorsuch and Ortberg [36], as attitudes serve as precondition “for someone to consider applying their learned knowledge or skills” [18].

As such, more attention should be given to attitudes as an important outcome measure for RCR education. However, studies investigating the effects of RCR training on attitudes have demonstrated mixed results [16,41,42]. For example, one study investigating the outcomes of an RCR course showed that the impact on knowledge was more significant than that for changes in skills or attitudes [16]. McGee and colleagues performed in-depth Interviews to study the effects of a course in RCR on the attitudes of doctorate and postdoctoral students. The impact of the course on attitudes was greater for students with limited prior knowledge in RCR compared to students who held prior experiences or existing knowledge that conflicted with what was taught [43]. Admitting, achieving a change in attitudes from RCR training programs can be variable among individuals, may be dependent of teachers’ skills [44], and may involve instruction that extends beyond just a few courses.

While attitudes are reflective of personal integrity, individuals’ perception of the integrity of their research environment as conveyed through knowledge of existing norms of behavior can also be instrumental in shaping proper research behaviors. Several studies have investigated such a relationship. For example, Hoffman and Holm surveyed postdoctoral researchers regarding their knowledge, attitudes and actions related to research misconduct as well as their perceptions of the integrity of the research environment [45]. These investigators demonstrated a “connection between attitudes and environmental integrity factors” [45]. In another study, Mumford and colleagues assessed the relationship between “ethical decision-making to climate and environmental experiences” in first-year doctoral students [46]. Aspects of the climate included “procedural justice, distributive justice, social context, individual caring, law and code, trust, freedom, and lack of conflict”. Environmental experiences included mentoring occurrences, production pressures, professional leadership, poor coping, lack of rewards, and poor career direction [46]. These investigators found that environmental experiences when compared with climate dimensions were better predictors of research integrity as determined by an “ethical decision-making measure” [46]. Overall, these studies suggest that research misbehaviors stem from personal integrity as well as influences from the environment in which individuals are situated [18].

We found positive results regarding correlations between prior ethics education and the attitude constructs. Holm and Hoffman demonstrated that previous ethics education was associated with lower RMSS [22] and Adeleye and Adebamowo found that ‘self-assessment of one’s knowledge of research ethics as being inadequate was associated with at least one type of research misconduct [47]. Other studies investigating the potential effects of ethics education on research misconduct have yielded mixed results [17,48–50]. Whether ethics training can be supportive of behaviors that reflect societal norms may be dependent on course design and length, pedagogy, the focus of the educational objectives, i.e., knowledge, skills, or attitudes, as well as the supporting environment.

### Limitations

There are several limitations in our study. First, after performing the reliability analysis and exploratory factor analysis, the general attitude factor consisted of only three items. Future efforts should expand this set of items by using the Delphi method that relies on a panel of experts. Second, as we used data obtained from individuals in the Arab Region, our attitude constructs may not be generalizable to other regions. Third, our data set was not large enough to add a confirmatory factor analysis. Finally, our data set was from 2015 and since this time there have been an increased focus on RCR resulting in additional training efforts and conferences. However, despite such efforts, the ability of measuring attitudes with a validated scale maintains its importance.

## Conclusions

Our study shows that the attitude scale adopted from our previous questionnaire study is a statistically valid and reliable tool for investigating constructs related to attitudes toward the acceptability of RCR practices as well as general attitudes regarding research misconduct. In developing educational programs in RCR as well as in survey research focused on research misconduct, it is important to be able to measure the attitudes of participants toward specific types of misconduct as well as their general attitudes toward misconduct. Results from such endeavors can help promote advances in the field of the responsible conduct in research.

## Supporting information

S1 File Table 1(a): Inter-item correlations of the “attitudes toward the acceptability of RCR practices” items. (DOCX)

S2 File Table 2 item-total correlation of the different items of the “attitudes toward the acceptability of RCR practices” and ‘‘general attitudes toward scientific misconduct” scales. (DOCX)
