# Supplementary material for: Reliability and validation of an attitude scale regarding responsible conduct in research
Source: PLoS One. 2022 Mar 16;17(3):e0265392. doi: 10.1371/journal.pone.0265392 (PMC8926210; doi:10.1371/journal.pone.0265392)
Supplement: S1 File — (DOCX) [file pone.0265392.s001.docx]

**Supplementary File S1**

Table 1(a): Inter-item correlations of the “attitudes toward the acceptability of RCR practices’’ items

|  |  |  |  |  |  |  |  |  |  |  |  |  |  |  |  |  |
| --- | --- | --- | --- | --- | --- | --- | --- | --- | --- | --- | --- | --- | --- | --- | --- | --- |
|  | RE_1 | RE_2 | RE_3 | DFF_1 | DFF_2 | DFF_3 | DFF_4 | Plag_1 | Plag_2 | Plag_3 | Authorship_1 | Authorship_2 | Authorship_3 | COI_1 | COI_2 | COI_3 |
| RE_1 | 1 | .699 | .751 | .677 | .692 | .586 | .604 | .669 | .635 | .613 | .596 | .657 | .643 | .681 | .683 | .709 |
| RE_2 |  | 1 | .847 | .730 | .755 | .695 | .655 | .756 | .762 | .671 | .653 | .746 | .709 | .710 | .719 | .719 |
| RE_3 |  |  | 1 | .699 | .758 | .669 | .689 | .691 | .702 | .670 | .624 | .674 | .663 | .680 | .689 | .705 |
| DFF_1 |  |  |  | 1 | .862 | .706 | .678 | .755 | .743 | .690 | .613 | .779 | .724 | .779 | .773 | .743 |
| DFF_2 |  |  |  |  | 1 | .784 | .753 | .811 | .812 | .737 | .702 | .807 | .753 | .767 | .796 | .801 |
| DFF_3 |  |  |  |  |  | 1 | .717 | .701 | .746 | .681 | .663 | .709 | .685 | .665 | .714 | .651 |
| DFF_4 |  |  |  |  |  |  | 1 | .635 | .690 | .669 | .671 | .635 | .670 | .635 | .688 | .675 |
| Plag_1 |  |  |  |  |  |  |  | 1 | .873 | .827 | .745 | .902 | .753 | .755 | .773 | .766 |
| Plag_2 |  |  |  |  |  |  |  |  | 1 | .844 | .730 | .851 | .770 | .771 | .770 | .765 |
| Plag_3 |  |  |  |  |  |  |  |  |  | 1 | .690 | .781 | .726 | .684 | .698 | .698 |
| Authorship_1 |  |  |  |  |  |  |  |  |  |  | 1 | .762 | .823 | .643 | .672 | .664 |
| Authorship_2 |  |  |  |  |  |  |  |  |  |  |  | 1 | .776 | .766 | .804 | .767 |
| Authorship_3 |  |  |  |  |  |  |  |  |  |  |  |  | 1 | .709 | .743 | .711 |
| COI_1 |  |  |  |  |  |  |  |  |  |  |  |  |  | 1 | .901 | .879 |
| COI_2 |  |  |  |  |  |  |  |  |  |  |  |  |  |  | 1 | .920 |

Table 1(b): Inter-item correlation of ‘’general attitudes toward research misconduct’’ items

|  | SM_1 | SM_3 | SM_4 | SM_5 |
| --- | --- | --- | --- | --- |
| SM_1 | 1 | .222 | .308 | .304 |
| SM_3 |  | 1 | .502 | .397 |
| SM_4 |  |  | 1 | .530 |

**Supplementary File S1**

Table 1(a): Inter-item correlations of the “attitudes toward the acceptability of RCR practices’’ items

|  |  |  |  |  |  |  |  |  |  |  |  |  |  |  |  |  |
| --- | --- | --- | --- | --- | --- | --- | --- | --- | --- | --- | --- | --- | --- | --- | --- | --- |
|  | RE_1 | RE_2 | RE_3 | DFF_1 | DFF_2 | DFF_3 | DFF_4 | Plag_1 | Plag_2 | Plag_3 | Authorship_1 | Authorship_2 | Authorship_3 | COI_1 | COI_2 | COI_3 |
| RE_1 | 1 | .699 | .751 | .677 | .692 | .586 | .604 | .669 | .635 | .613 | .596 | .657 | .643 | .681 | .683 | .709 |
| RE_2 |  | 1 | .847 | .730 | .755 | .695 | .655 | .756 | .762 | .671 | .653 | .746 | .709 | .710 | .719 | .719 |
| RE_3 |  |  | 1 | .699 | .758 | .669 | .689 | .691 | .702 | .670 | .624 | .674 | .663 | .680 | .689 | .705 |
| DFF_1 |  |  |  | 1 | .862 | .706 | .678 | .755 | .743 | .690 | .613 | .779 | .724 | .779 | .773 | .743 |
| DFF_2 |  |  |  |  | 1 | .784 | .753 | .811 | .812 | .737 | .702 | .807 | .753 | .767 | .796 | .801 |
| DFF_3 |  |  |  |  |  | 1 | .717 | .701 | .746 | .681 | .663 | .709 | .685 | .665 | .714 | .651 |
| DFF_4 |  |  |  |  |  |  | 1 | .635 | .690 | .669 | .671 | .635 | .670 | .635 | .688 | .675 |
| Plag_1 |  |  |  |  |  |  |  | 1 | .873 | .827 | .745 | .902 | .753 | .755 | .773 | .766 |
| Plag_2 |  |  |  |  |  |  |  |  | 1 | .844 | .730 | .851 | .770 | .771 | .770 | .765 |
| Plag_3 |  |  |  |  |  |  |  |  |  | 1 | .690 | .781 | .726 | .684 | .698 | .698 |
| Authorship_1 |  |  |  |  |  |  |  |  |  |  | 1 | .762 | .823 | .643 | .672 | .664 |
| Authorship_2 |  |  |  |  |  |  |  |  |  |  |  | 1 | .776 | .766 | .804 | .767 |
| Authorship_3 |  |  |  |  |  |  |  |  |  |  |  |  | 1 | .709 | .743 | .711 |
| COI_1 |  |  |  |  |  |  |  |  |  |  |  |  |  | 1 | .901 | .879 |
| COI_2 |  |  |  |  |  |  |  |  |  |  |  |  |  |  | 1 | .920 |

Table 1(b): Inter-item correlation of ‘’general attitudes toward research misconduct’’ items

|  | SM_1 | SM_3 | SM_4 | SM_5 |
| --- | --- | --- | --- | --- |
| SM_1 | 1 | .222 | .308 | .304 |
| SM_3 |  | 1 | .502 | .397 |
| SM_4 |  |  | 1 | .530 |
