# Supplementary material for: Reliability and validation of an attitude scale regarding responsible conduct in research
Source: PLoS One. 2022 Mar 16;17(3):e0265392. doi: 10.1371/journal.pone.0265392 (PMC8926210; doi:10.1371/journal.pone.0265392)
Supplement: S2 File — (DOCX) [file pone.0265392.s002.docx]

**Supplementary File S2**

**Table 2** Item-total correlation of the different items of the “attitudes toward the acceptability of RCR practices’’ and ‘’general attitudes toward scientific misconduct’’ scales

| **Variable** | **Item-total correlation** |
| --- | --- |
| Attitudes toward acceptability of RCR practices |  |
| RE_2 | 0.86 |
| RE_3 | 0.84 |
| DFF_1 | 0.87 |
| DFF_2 | 0.91 |
| DFF_3 | 0.83 |
| DFF_4 | 0.81 |
| Plag_1 | 0.90 |
| Plag_2 | 0.90 |
| Plag_3 | 0.85 |
| Authorship_1 | 0.82 |
| Authorship_2 | 0.90 |
| Authorship_3 | 0.86 |
| COI_1 | 0.87 |
| COI_2 | 0.89 |
| COI_3 | 0.88 |
| General attitudes toward scientific misconduct |  |
| SM_1 | 0.64 |
| SM_3 | 0.72 |
| SM_4 | 0.78 |
| SM_5 | 0.78 |
